# IN-MACA-MCC: Integrated Multiple Attractor Cellular Automata with Modified Clonal Classifier for Human Protein Coding and Promoter Prediction

**DOI:** 10.1155/2014/261362

**Published:** 2014-07-15

**Authors:** Kiran Sree Pokkuluri, Ramesh Babu Inampudi, S. S. S. N. Usha Devi Nedunuri

**Affiliations:** ^1^Department of CSE, JNTU, Hyderabad 500 085, India; ^2^Department of CSE, Acharya Nagarjuna University, Guntur 522510, India; ^3^Department of CSE, University College of Engineering, JNTU, Kakinada 533003, India

## Abstract

Protein coding and promoter region predictions are very important challenges of bioinformatics (Attwood and Teresa, 2000). The identification of these regions plays a crucial role in understanding the genes. Many novel computational and mathematical methods are introduced as well as existing methods that are getting refined for predicting both of the regions separately; still there is a scope for improvement. We propose a classifier that is built with MACA (multiple attractor cellular automata) and MCC (modified clonal classifier) to predict both regions with a single classifier. The proposed classifier is trained and tested with Fickett and Tung (1992) datasets for protein coding region prediction for DNA sequences of lengths 54, 108, and 162. This classifier is trained and tested with MMCRI datasets for protein coding region prediction for DNA sequences of lengths 252 and 354. The proposed classifier is trained and tested with promoter sequences from DBTSS (Yamashita et al., 2006) dataset and nonpromoters from EID (Saxonov et al., 2000) and UTRdb (Pesole et al., 2002) datasets. The proposed model can predict both regions with an average accuracy of 90.5% for promoter and 89.6% for protein coding region predictions. The specificity and sensitivity values of promoter and protein coding region predictions are 0.89 and 0.92, respectively.

## 1. Introduction

Many of the important problems [1] in bioinformatics can beaddressed with our computing techniques very easily. So we have identified two major problems in bioinformatics and worked on them basically to understand the logicalities in these two problems. After an extensive literature survey we have developed the frame work for addressing these problems. This frame work developed can be useful for addressing other problems in bioinformatics like splice junction prediction, secondary structure prediction of protein, and so forth. The proposed (IN-MACA-MCC) classifier can predict both promoter and protein coding regions very easily with more accuracy when compared with existing literature with less time.

DNA is an important component of a cell and genes will be found in specific portion of DNA which will contain the information as explicit sequences of bases (A, G, C, and T). These explicit sequences of nucleotides will have instructions to build the proteins. But the region which will have the instructions which is called protein coding regions occupies very less space in a DNA sequence. The identification of protein coding regions plays a vital role in understanding the genes. We can extract lot of information like what is the disease causing gene, whether it is inherited from father or mother and a promoter can regulate the growth of disease slowly, and how one cell is going to control another cell. Although the entire human genome is sequenced, identifying the protein coding region as well as finding the gene is still a complicated process.

DNA contains lots of information. We need promoter for DNA transcription to from RNA. So promoter plays a vital role in DNA transcription. It is defined as “the sequence in the region of the upstream of the transcriptional start site (TSS).” Identifying a new promoter in a DNA sequence will lead to finding a new protein. If we identify the promoter region we can extract information regarding gene expression patterns, cell specificity, and development. Promoters will regulate a gene expression. Some of the genetic diseases which are associated with variations in promoters are asthma, beta thalassemia, and Rubinstein-Taybi syndrome. Promoter sequence can be used to control the speed of translation from DNA into protein. It is also used in genetically modified foods.

In vertebrates only five percent of the gene is made up of exons. Genes mostly will have seven to eight exons with 145 bp length at an average. Introns have 3365 bp length at an average. Promoter comprises a small percentage of entire genome. The features of promoters are different from other functional regions like exons, introns, and 3′UTRs. These facts make protein coding and promoter region predictions very difficult tasks.

This paper is organized in the following manner.  Section 2 provides the entire literature survey of both protein coding and promoter regions.  Section 3 provides the design of the proposed system.  Section 4 presents the MACA-MCC classifier for promoter and protein coding prediction.  Section 5 provides the experimental results with discussion.  Section 6 provides the future extensions and conclusion to the proposed classifier.

## 2. Literature Survey

Salzberg has used a decision tree algorithm [2] for locating protein coding regions in DNA sequences, which is adaptable and can process DNA sequences of lengths 54 bp, 108 bp and 162 bp. Maji and Paul [3] have developed neural network tree classifier for prediction of splice junction and coding regions in genomic DNA. A decision tree named NNTree (neural network tree) is constructed by dividing the training set with their corresponding labels to recursively generate a tree. Xu et al. [4] have developed an improved system GRAIL II which is a hybrid AI system which can predict the number of exons in a human DNA sequence and also supports gene modeling. This process combines edge signal like accepter, donor, translation start site detection, and coding feature analysis.

Snyder and Stormo [5] have applied dynamic programming and neural networks for predicting protein coding regions from a genomic DNA. They have developed a program GeneParser which first scores the DNA sequences based on exon-intron specific measures like local compositional complexity, codon usage, length distribution, 6-tuple frequency, and periodic asymmetry. Uberbacher and Mural [6] have proposed a method which combines some set of sensor algorithms and neural network to predict the protein coding regions in eukaryotes. The programs developed will calculate the values of seven sensors that were considered by the authors. The measures are frame bias matrix, Fickett (three-periodicity), dinucleotide fractal dimension, coding six tuple word preferences, coding six tuples in frame preferences, word commonality, and repetitive six tuple word preferences.

Pinho et al. [7] have proposed a three-state model for protein coding region prediction. Authors have considered three-base periodicity property. Zhang [8] has used quadratic discriminant analysis method named MZEF for identifying protein coding regions in genomic human DNA. Gish and States [9] proposed a computer program named BLASTC which uses sequence similarity and codon utilization for predicting the protein coding regions.

Method in [3] takes more time to construct a tree for sequences of length 162. The height of the trees is also a major concern for using this algorithm with DNA sequences of more length. Method in [4] suffers with less accuracy due to more error rate at classifier nodes. Methods in [5–7] depend more on the statistical information. After this literature survey the concern of a new classifier is to achieve good classifier accuracy and develop a classifier which can handle DNA sequences of length more than 162 with fewer nodes.

Zeng et al. [10] have proposed a hierarchical promoter prediction system named SCS where they have used signal, structure, and context features. Li et al. [11] have proposed a method PCA-HPR (principal component analysis-human promoter recognition) to predict the promoters and transcription sites (TSS). Hannenhalli and Levy [12] tried to enhance the accuracy of promoter prediction by combining CpG island feature with information of independent signals which are biologically motivated and these cover most of the knowledge to predict the promoter in human genome.

Wu et al. have proposed a method [13] for enhancing the performance of human promoter region identification by selecting the most important features of DNA sequence for each different functional region. Ohler et al. have proposed a model [14] which integrates physical properties of DNA into a probabilistic eukaryotic promoter prediction system. Goñi et al. have proposed a system ProStar [15] which uses structural parameters for promoter region identification. Authors only used descriptors derived from physical first principles.

Bajic et al. [16] have developed new software for identifying promoters in a DNA sequence of vertebrates. This program takes input as DNA sequence and generates a list of predicted TSS (transcription stating site). Zhang [17] has proposed a new program for predicting a core promoter in human gene named as CorePromoter. After the literature survey on promoter prediction, the main goal of proposed classifier is to reduce the false prediction rates and improve specificity and sensitivity values.

## 3. Design of IN-MACA-MCC

**Figure 1:**
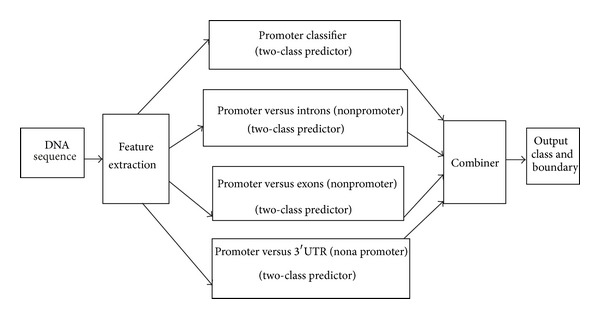
IN-MACA-MCC architecture—front.

**Figure 2:**
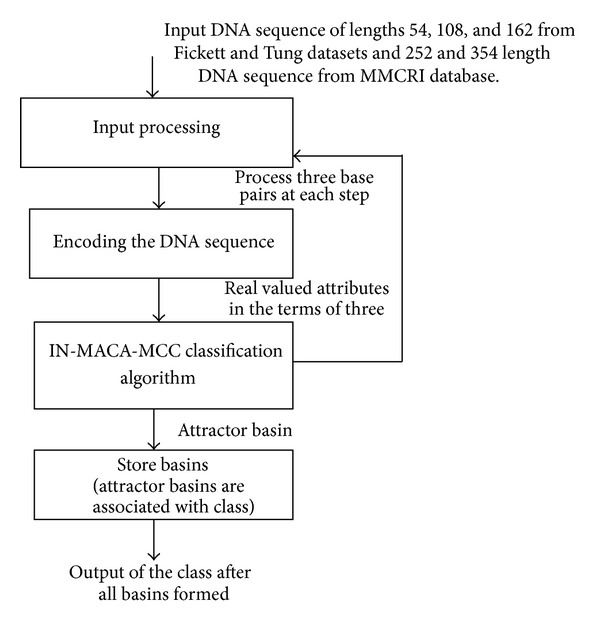
IN-MACA-MCC architecture—rear.

IN-MACA-MACC basic processing as shown in Figure 1 starts with identification promoter considering features like TATA, CAAT, Inr, and n-mers unlike AIX-MACA-Y [18], for predicting both regions. IN-MACA-MCC takes a DNA input and checks whether it belongs to a promoter or not. If it belongs to promoter the exact boundaries are provided. If the given input is a nonpromoter sequence it checks whether it belongs to intron or exon or 3′UTR. If it belongs to an exon IN-MACA-MCC reports the boundaries of the first exon. These boundaries will be used by the next module as shown in Figure 2 to trace the protein coding region starting from that boundary. If the input does not belong to exons, it will check with introns and 3′UTRs and outputs the class accordingly.

The design rear IN-MACA-MACC is indicated in Figure 2. Input to IN-MACA-MACC algorithm and its variations will be DNA sequence and amino acid sequences. Input processing unit will process sequences three at a time as three neighborhood cellular automata are considered for processing DNA sequences. The rule generator will transform the complemented and noncomplemented rules in the form of matrix, so that we can apply the rules to the corresponding sequence positions very easily. IN-MACA-MACC basins are calculated as per the instructions of proposed algorithm.

Cellular automata that use fuzzy logic are an array of cells arranged in linear fashion evolving with time. Every cell of this array assumes a rational value in the interval of zero and one. All these cells change their states according to the local evaluation function which is a function of its state and its neighboring states. The synchronous application of the local rules to all the cells of array will depict the global evolution.  Table 1 shows some rules for developing the proposed classifier.

**Example 1 .**

Consider the rule 〈170, 238, 204〉 and corresponding transition matrix is shown below.

If *P*(0) is the initial state with real values (0, 0.25, 0.50) the successive three steps are defined below.

The transitions from one state to another state are defined as

(1) $$\begin{array}{c} T=\left[\begin{array}{ccc} 0 & 1 & 0\\ 0 & 1 & 1\\ 0 & 0 & 1 \end{array}\right]\\ P\left(0\right)=\left(0,0.25,0.50\right). \end{array}$$

**Step 1 .**

Apply rule 170 for the first cell. Rule  170 says that the next state depends on the right neighbor. Consider

(2) $$\begin{array}{c} P\left(1\right)=\left(0.25,0.25,0.50\right). \end{array}$$

Apply rule 238 to the second cell. Rule  238 says that the next state depends on its state and the right neighbor. Consider

(3) $$\begin{array}{c} P\left(1\right)=\left(0.25,0.75,0.50\right)\;\left(0.25+0.50\right). \end{array}$$

Apply rule 204 to the third cell. Rule  204 says that the next state depends only on its state.

After applying the rule for all the cells in the state is (0.25, 0.75, 0.50) that is the resultant state after first iteration

(4) $$\begin{array}{c} P\left(1\right)=\left(0.25,0.75,0.50\right). \end{array}$$

Similarly, one has the following.

**Step 2 .**

Consider.

(5) $$\begin{array}{c} P\left(2\right)=\left(0.75,1.0,0.50\right). \end{array}$$

**Step 3 .**

Consider.

(6) $$\begin{array}{c} P\left(3\right)=\left(1.0,1.0,0.50\right). \end{array}$$

**Figure 3:**
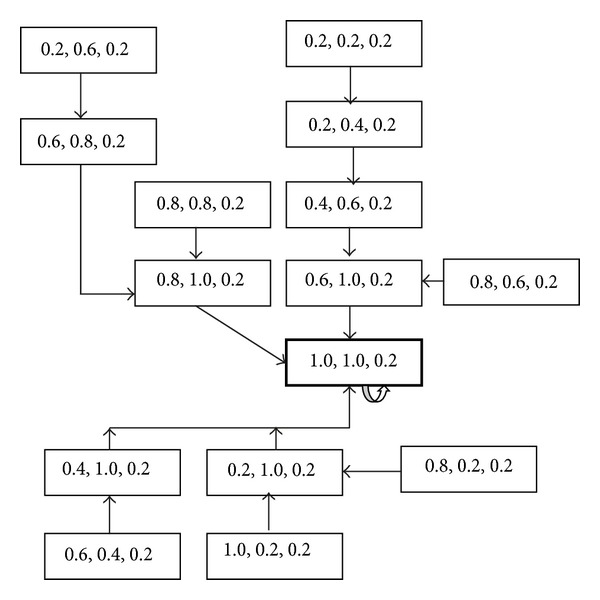
Attractor state (1.0, 1.0, and 0.2)—B formed with rule 〈170, 252, 204〉.

Likewise we can construct IN-MACA-MCC for a sample dataset as shown in Figure 3.

## 4. Modified Clonal Classifier with MACA

### 4.1. Simplified Modified Clonal Algorithm

1. Generate initial antibody population (AIS-MACA rules) randomly and call it Ab. It consists of two subsets memory population Ab_*m*_ and reservoir population Ab_*r*_.
2. Construct a set of antigens population and call it Ag (DNA sequence with class/input).
3. Select an antigen Ag_*j*_ from Ag the antigen population.
4. Apply every member of antibody population to the selected antigen Ag_*j*_, check whether it is predicting the correct class, and calculate affinity of the rule with the antigen via fitness equation.
5. Select *m* highest affinity antibodies (AIS-MACA rules) from Ab and place them in *P*_*m*_.
6. Generate clones for each antibody, which will be proportional to the affinity as per fitness. Place the clones in the new population *P*_*i*_.
7. Apply mutation to the newly formed population *P*_*i*_ where the degree is inversely proportional to their affinity. This produces a more mature population *P*_i_*.
8. *R*_*e*_ calculate the affinity of the rule with the corresponding antigen as we did it in step four. Order the antibodies in descending order (high fitness antibody will be on top).
9. Compare the antibodies from *P*_i_* with the antibodies population from Ab_*m*_. Select the better fitness rules, remove them from *P*_i_*, and place them in Ab_*m*_.
10. Randomly generate antibodies for introducing diversity. Compare the antibodies in Ab_*r*_, the left-out antibodies in *P*_i_*, and randomly generate antibodies. Select the better fitness rules among three and place them in Ab_*r*_.
11. For every generation, compare the antibodies in Ab_*m*_ and Ab_*r*_ and place the best in Ab_*m*_.

### 4.2. Difference between Clonal and Modified Clonal Algorithm

The difference between original clonal algorithm and the modified algorithm proposed by us lies on how efficiently we are managing the use of generated antibodies. Original clonal algorithm will not take advantage of the antibodies generated by every cloned population. Once the comparison of antibodies in *P*_i_* and Ab_*m*_ gets completed, the best will be placed in Ab_*m*_ and the rest of antibodies in *P*_i_* are omitted. Even the reservoir antibodies are poorly maintained. So we try to use the best antibodies in *P*_i_* left out after placing them in Ab_*m*_. For this purpose we are comparing the antibodies already in Ab_*r*_ with left-out antibodies in *P*_i_* and newly generated antibodies which were meant for introducing diversity. After comparing the three sets the best will be placed in Ab_*r*_. In the original clonal algorithm step 11 will not exit. Step 11 will ensure the best fitness rules stay in Ab_*m*_ which will be solution of the entire problem.

## 5. Experimental Results

**Figure 4:**
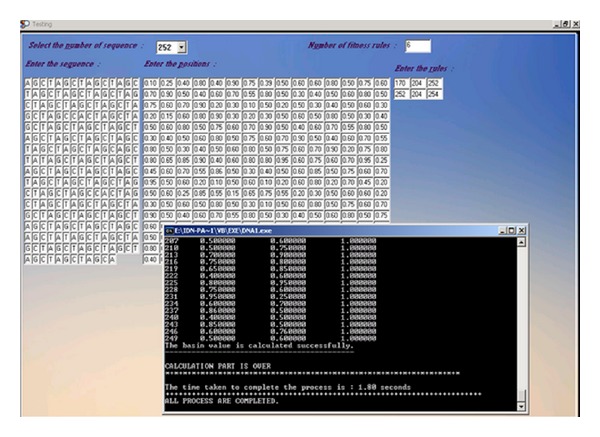
Basin calculation.

**Figure 5:**
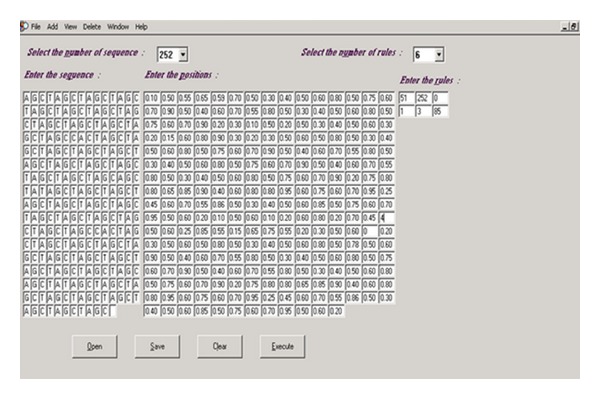
Training interface.

**Figure 6:**
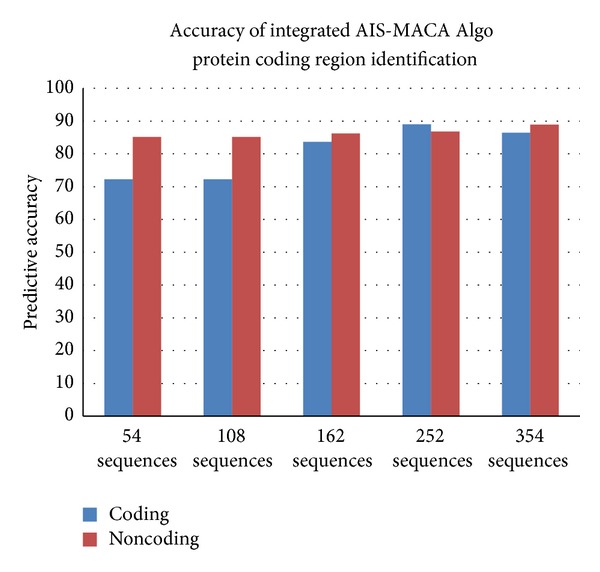
Predictive accuracy for protein coding regions.

**Figure 7:**
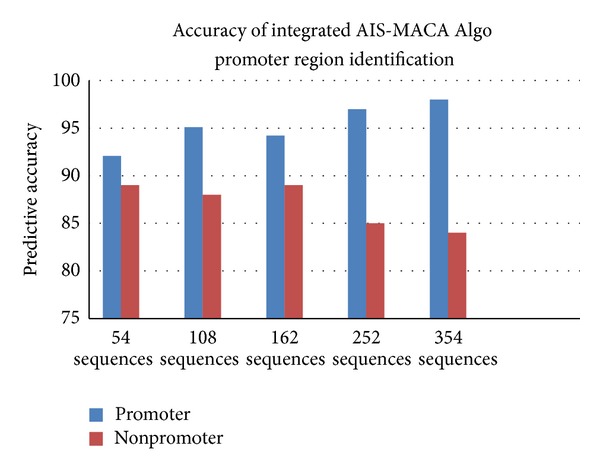
Predictive accuracy for promoter regions.

The proposed classifier is trained and tested with Fickett and Tung [19] datasets for protein coding region prediction for DNA sequences of lengths 54, 108, and 162. All the 21 measures reported in [19] were considered for developing the classifier. This classifier is trained and tested with MMCRI (http://www.mmchri.res.in/) [20] datasets for protein coding region prediction for DNA sequences of lengths 252 and 354. The proposed classifier is trained and tested with promoter sequences from DBTSS [21] dataset and nonpromoters from EID [22] and UTRdb [23] datasets. Figures 4 and 5 show the developed interfaces.  Table 2 shows the execution time for predicting both protein and promoter regions which is very promising. Tables 3 and 4 show the sensitivity and specificity of both predictions. All the experiments are performed in SUN with Solaris 5.8, 445 MHz clock. Figures 6 and 7 show the accuracy of prediction separately which is the important output of our work.

### 5.1. Specific Output of 54 Length DNA Sequence with Boundaries

See Box 1, Figures 4, 5, 6, and 7, and Table 2.

### 5.2. Human Promoter Output

See Box 2 and Tables 3 and 4.

## 6. Conclusion

We have successfully developed a classifier which can predict promoter and protein coding regions with higher accuracy than reported earlier. The sensitivity and specificity values for both predictions are also promising. There is considerable improvement in the reduction of false prediction rate. IN-MACA-MCC attains highest accuracy of 92.3% for sequences more than 108 bp and less than 552 bp for protein coding region prediction. IN-MACA-MCC attains highest accuracy of 93.6% for sequences of length 251 for promoter regions. We are trying to apply this classifier for most of the species in eukaryotes in future.

## Figures and Tables

**Box 1:**
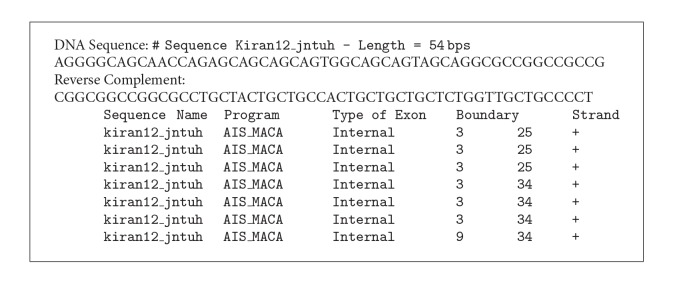


**Box 2:**
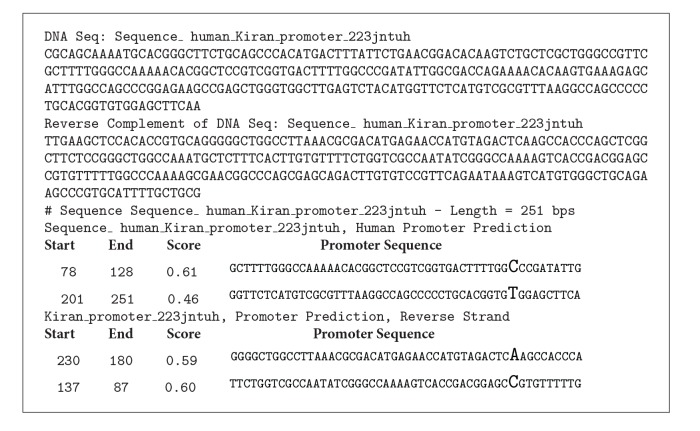


**Table 1:** Example rules.

SNO	Rule number	General representation
1	254	*q*_*i*−1_ + *q*_*i*_ + *q*_*i*+1_
2	252	*q*_*i*−1_ + *q*_*i*_
3	238	*q*_*i*_ + *q*_*i*+1_
4	250	*q*_*i*−1_ + *q*_*i*+1_
5	204	*q*_*i*_
6	240	*q*_*i*−1_
7	170	*q*_*i*+1_

**Table 2:** Execution time for prediction of both protein and promoter regions.

Size of dataset	Prediction time of integrated algorithm in ms
5000	1064
6000	1389
10000	2002
20000	2545

**Table 3:** IN-MACA-MCC protein coding comparison with existing approaches.

Algorithm/coding measure	Sensitivity	Specificity
OC1	65.3	66.4
Hexamer	68.36	70.2
Position asymmetry	72.3	74.5
Dicodon usage	81.3	82.3
CRITICA	82.5	84.9
IN-MACA-MCC	89.6	89.3

**Table 4:** IN-MACA-MCC promoter comparison with existing approaches.

Method	Sensitivity	Specificity
Promoter inspector	56.9	46.9
Dragon promoter finder	62.3	59.3
Promo predictor	65.3	66.9
CNN-promoter	76.3	82.3
SPANN	68.9	84
IMC	76	86
IN-MACA-MCC	88.5	92.7
